# Machine learning model for early prediction of acute respiratory failure in acute pancreatitis: Multicenter validation

**DOI:** 10.1016/j.isci.2026.116470

**Published:** 2026-06-18

**Authors:** Yu Wang, Feng Lv, MingYang Tao, JiXuan Cui, Dan Zhang, Haodong Zhao, Jun Li, GengYun Sun, XingYu Wang

**Affiliations:** 1 Department of Emergency Medicine, The First Affiliated Hospital of Anhui Medical University, Hefei City, Anhui Province 230001, China; 2 Hefei Urban Rail Transit Group Co., Ltd, Hefei City, Anhui Province 230001, China; 3 Department of Respiratory and Critical Care Medicine, The First Affiliated Hospital of Anhui Medical University, Hefei City, Anhui Province 230001, China; 4 School of Electronic and Optical Engineering, Nanjing University of Science and Technology, Nanjing City, Jiangsu Province 210094, China; 5 School of Information Science and Engineering, Southeast University, Nanjing City, Jiangsu Province 211189, China

**Keywords:** Health sciences

## Abstract

Acute respiratory failure (ARF) is a common organ failure in acute pancreatitis (AP) with high mortality. This study used machine learning to predict ARF risk in AP patients through a retrospective, multicenter cohort analysis involving MIMIC-IV and Chinese hospital data (2010–2025). Variable selection combined SHapley Additive exPlanations values and Lasso regression, employing five machine learning algorithms for modeling. The dataset identified nine most influential features, including temperature, SpO_2_, and seven serum indicators, with the logistic regression (LR) model achieving the highest areas under the curve (AUCs) (0.9048 internal, 0.8717 external, and 0.8557 cross-specialty) and top sensitivity (0.8947 internal, 0.7361 external, and 0.9 cross-specialty). The LR model proved to be the most effective classifier for early ARF prediction in AP. A cross-specialty validation of pregnancy patients delineated the model’s boundary condition. An online computing platform for this LR model was publicly available and free to use.

## Highlights

• A model to predict ARF in AP was developed and validated using a spatiotemporal framework
• The study identified a key performance boundary through a cross-specialty validation of APIP
• An easy-to-access website calculator of the LR model was constructed for clinicians

## Graphical abstract

**Figure:**
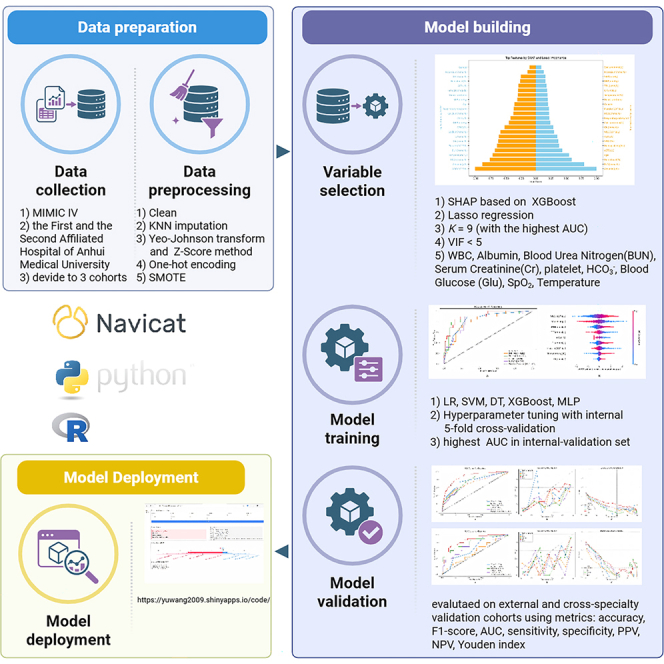

## Introduction

Acute pancreatitis (AP) is an inflammatory disease of the pancreas, with the high incidence rate of 33.74 per 100,000 person-years and mortality of 1.60 deaths per 100,000 person-years.^1^ Approximately 20% of patients develop moderate-to-severe pancreatitis, which may involve single or multiple organ failure and life-threatening conditions.^2^ Acute respiratory failure (ARF) is the most common type of organ failure in AP, with high incidence and mortality rates.^3^^,^^4^ The respiratory system is often the first to fail and the last to recover. Mortality in patients with necrotizing pancreatitis is 15%, which increases to 35% when complicated by organ failure.^5^ This has a significant impact on patient prognosis. Consequently, early identification and prediction of the risk of ARF in AP, along with appropriate ventilatory management, are crucial to improving patient outcomes.

Currently, the Acute Lung Injury Score (LIPS) is a widely used model for predicting acute respiratory distress syndrome (ARDS) in at-risk patients; however, the original development cohort for this model consisted of only a small number of patients with AP.^6^ Although Li’s study reported that LIPS could predict respiratory failure in acute pancreatitis, the training and validation cohorts were exclusively derived from tertiary hospitals in China.^7^ The widespread application of early predictive models for ARF in AP in clinical practice still faces many challenges.

The mechanisms of direct pulmonary injury in AP are primarily related to inflammatory mediators secreted by the pancreas and their effects on pulmonary vasculature. ARDS is recognized as a major cause of respiratory failure in AP, with a high mortality rate.^8^ Moreover, patients with acute pancreatitis often experience intestinal dysfunction, and the translocation of gut bacteria and endotoxins further exacerbates pulmonary inflammation, forming a “gut-lung axis” injury mechanism.^9^ Elevated intra-abdominal pressure in acute pancreatitis is considered a risk factor for respiratory failure.^10^ However, due to the complexity and diversity of these indicators, the sensitivity and specificity of individual indicators are limited. To address these gaps, this study aimed to develop a parsimonious and accurate predictive model for early-stage ARF in AP patients, prioritizing clinical interpretability and operational feasibility.

With the rapid development of big data and artificial intelligence technologies, predictive models based on personalized data have shown great potential in the risk assessment, treatment selection, and prognosis prediction of AP.^11^^,^^12^ Our study integrated large-scale from multicenter databases across two countries to develop an artificial-intelligence-based predictive model. The easily accessible variables included demographics, laboratory indicators, and vital signs at admission. Through continuous optimization and validation, this predictive model is expected to play a larger role in clinical practice and help reduce ventilator use and mortality rates, thereby alleviating the health burden on AP patients.

## Results

### Patient characteristics

**Figure 1:**
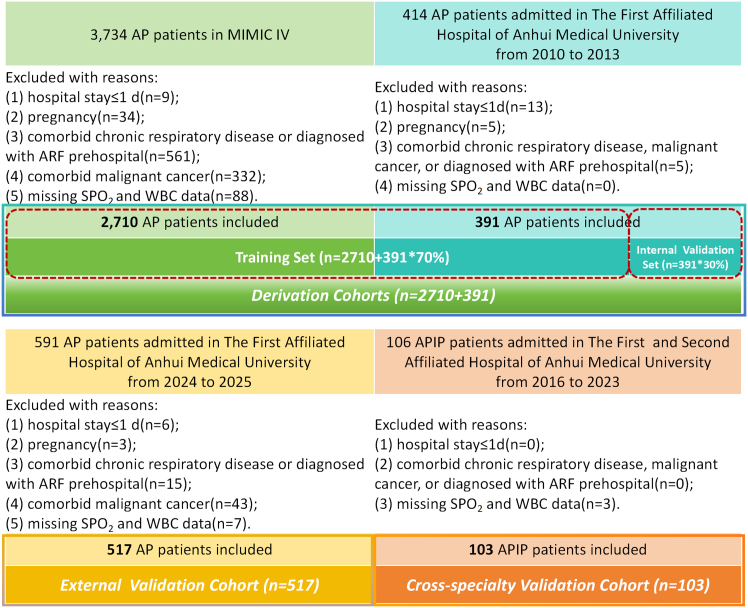
Study participant selection

In this study, 3,101 patients with AP formed the derivation cohort from the Medical Information Mart for Intensive Care IV (MIMIC IV)^13^ and the First Affiliated Hospital of Anhui Medical University in China, and 517 patients from the same hospital in China constituted the external validation cohort. In addition, 103 patients with acute pancreatitis in pregnancy (APIP) from the First Affiliated Hospital of Anhui Medical University and the Second Affiliated Hospital of Anhui Medical University were extracted as a cross-specialty validation cohort (Figure 1).

Among 3,101 AP patients, 303 (9.8%) developed ARF in the derivation cohort and 72 (13.9%) in the external cohort. The ventilator usage was 15.8% in the derivation cohort and 2.1% in the external cohort. Thirty of one hundred three (29.1%) pregnant patients with AP developed ARF in cross-specialty validation, and ventilator usage was 27.2%. During follow-up, 20.3% of the derivation cohort and 7.2% of the external cohort required intensive care unit (ICU) admission, with in-hospital mortality rates of 2.1% and 1.0%, respectively. In the cross-specialty validation cohort, 45.6% of patients required ICU involvement, with no cases of in-hospital maternal mortality, and the fetal loss rate was 2.9%. Patient characteristics are detailed in Table 1.

**Table 1:** The baseline characteristics of each cohort

Variables	Total (*n* = 3,721)	Derivation cohort (*n* = 3,101)	External validation cohort (*n* = 517)	Cross-specialty validation cohort (*n* = 103)	*p* value
Race, n (%)					<0.001
ASIAN	1101 (29.6)	481 (15.5)	517 (100)	103 (100)	
BLACK	318 (8.5)	318 (10.3)	0 (0)	0 (0)	
HISPANIC	148 (4.0)	148 (4.8)	0 (0)	0 (0)	
OTHER/UNKNOWN	275 (7.4)	275 (8.9)	0 (0)	0 (0)	
WHITE	1,879 (50.5)	1,879 (60.6)	0 (0)	0 (0)	
Gender, n (%)					<0.001
F	1,788 (48.1)	1,476 (47.6)	209 (40.4)	103 (100)	
M	1,933 (51.9)	1,625 (52.4)	308 (59.6)	0 (0)	
Age (years)	54.0 (41.0, 68.0)	56.0 (44.0, 69.0)	47.0 (36.0, 61.0)	29.0 (26.0, 33.0)	<0.001
Heart rate (b/m)	87.0 (76.0, 100.0)	85.0 (74.0, 98.0)	89.0 (78.0, 102.0)	111.0 (98.0, 125.0)	<0.001
SBP (mmHg)	129.0 (114.0, 144.0)	129.0 (113.0, 145.0)	130.0 (118.0, 142.0)	116.5 (107.0, 129.0)	<0.001
DBP (mmHg)	76.0 (67.0, 86.0)	75.0 (65.0, 85.0)	80.0 (72.0, 89.0)	75.0 (68.0, 81.8)	<0.001
MAP (mmHg)	93.7 (83.3, 104.3)	93.3 (82.3, 104.3)	96.3 (88.3, 106.0)	88.6 (81.8, 97.0)	<0.001
Temperature (°C)	36.8 (36.5, 37.1)	36.8 (36.6, 37.1)	36.5 (36.5, 36.8)	37.1 (36.6, 37.8)	<0.001
Respiratory rate (b/m)	18.0 (16.0, 20.0)	18.0 (16.0, 20.0)	20.0 (19.0, 20.0)	21.0 (20.0, 25.0)	<0.001
SpO_2_ (%)	98.0 (97.0, 100.0)	98.0 (96.0, 100.0)	99.0 (98.0, 100.0)	98.0 (96.0, 99.0)	<0.001
WBC (10^9^/L)	10.1 (7.0, 13.9)	9.7 (6.7, 13.6)	11.6 (8.1, 14.8)	13.2 (11.0, 16.6)	<0.001
Neutrophil (10^9^/L)	9.4 (6.1, 13.1)	9.2 (5.9, 13.3)	9.5 (6.0, 12.5)	11.6 (9.2, 15.2)	<0.001
Lymphocyte (10^9^/L)	1.2 (0.7, 1.7)	1.1 (0.7, 1.6)	1.4 (0.9, 1.8)	1.0 (0.8, 1.3)	<0.001
NLR (%)	8.3 (4.2, 14.9)	8.6 (4.2, 15.8)	6.8 (3.7, 11.9)	12.1 (7.4, 17.5)	<0.001
Platelet (10^9^/L)	208.0 (156.0, 267.0)	206.0 (154.0, 268.0)	213.0 (166.0, 260.0)	204.0 (163.0, 279.0)	0.52
Hematocrit (%)	37.0 (33.0, 41.4)	36.4 (32.7, 40.7)	40.9 (36.8, 44.8)	35.0 (31.0, 37.8)	<0.001
Hemoglobin (g/dL)	124.0 (110.0, 140.0)	122.0 (108.0, 137.0)	142.0 (125.0, 158.0)	120.5 (110.0, 133.0)	<0.001
Albumin (g/L)	36.0 (30.3, 41.2)	35.0 (30.0, 40.0)	41.0 (36.8, 44.7)	31.0 (26.4, 36.4)	<0.001
ALT (U/L)	48.0 (23.0, 160.0)	55.0 (24.0, 177.0)	34.0 (21.0, 84.8)	24.0 (15.5, 41.0)	<0.001
AST (U/L)	50.0 (26.0, 140.2)	58.0 (27.0, 152.0)	34.0 (23.0, 80.0)	28.0 (20.0, 47.0)	<0.001
LDH (U/L)	276.0 (188.0, 522.8)	312.0 (192.0, 611.5)	236.5 (182.2, 328.2)	256.0 (183.0, 352.5)	<0.001
TBIL (μmol/L)	17.1 (10.3, 33.8)	16.7 (8.6, 34.3)	20.0 (14.2, 30.8)	13.9 (10.2, 22.9)	<0.001
Cr (μmol/L)	70.7 (53.0, 88.4)	70.7 (58.0, 97.2)	63.0 (51.6, 75.7)	46.8 (36.3, 55.0)	<0.001
BUN (mmol/L)	5.0 (3.2, 7.1)	5.0 (3.2, 7.5)	5.1 (3.8, 6.5)	3.1 (2.0, 4.2)	<0.001
Calcium (mmol/L)	2.1 (2.0, 2.2)	2.1 (2.0, 2.2)	2.1 (2.0, 2.2)	2.1 (1.8, 2.2)	0.005
Potassium (mmol/L)	3.9 (3.7, 4.3)	3.9 (3.6, 4.3)	4.0 (3.8, 4.3)	3.9 (3.6, 4.2)	<0.001
Sodium (mmol/L)	139.0 (136.0, 141.0)	139.0 (136.0, 141.0)	138.4 (135.2, 140.4)	135.0 (131.3, 137.9)	<0.001
Glu (mmol/L)	6.4 (5.1, 8.4)	6.2 (5.1, 8.1)	7.4 (6.1, 10.0)	6.5 (4.9, 8.7)	<0.001
TCH (mmol/L)	4.6 (3.5, 6.3)	4.2 (3.2, 5.4)	4.8 (3.8, 6.7)	7.6 (5.3, 12.9)	<0.001
TG (mmol/L)	1.5 (1.0, 3.9)	1.4 (0.9, 2.5)	2.7 (1.1, 6.0)	5.6 (2.7, 19.5)	<0.001
HCO_3_ (mmol/L)	24.0 (21.0, 26.0)	24.0 (21.6, 26.0)	23.5 (19.4, 26.5)	17.0 (12.9, 20.0)	<0.001
Hospitalization days	5.6 (3.1, 9.8)	4.9 (2.9, 9.2)	7.0 (5.0, 10.0)	11.0 (7.0, 16.0)	<0.001
Acute respiratory failure n (%)					<0.001
0	3,316 (89.1)	2,798 (90.2)	445 (86.1)	73 (70.9)	
1	405 (10.9)	303 (9.8)	72 (13.9)	30 (29.1)	
Ventilation, n (%)					<0.001
0	3,192 (85.8)	2,611 (84.2)	506 (97.9)	75 (72.8)	
1	529 (14.2)	490 (15.8)	11 (2.1)	28 (27.2)	
ICU, n (%)					<0.001
0	3,009 (80.9)	2,473 (79.7)	480 (92.8)	56 (54.4)	
1	712 (19.1)	628 (20.3)	37 (7.2)	47 (45.6)	
In-hospital mortality n (%)					0.084
0	3,651 (98.1)	3,036 (97.9)	512 (99)	103 (100)	
1	70 (1.9)	65 (2.1)	5 (1)	0 (0)	

Abbreviations: SBP, systolic blood pressure; DBP, diastolic blood pressure; MAP, mean arterial pressure; WBC, white blood cell; NLR, neutrophil/lymphocyte ratio; Hct, hematocrit; TBIL, total bilirubin; ALT, alanine aminotransferase; AST, aspartate aminotransferase; Cr, serum creatinine; BUN, blood urea nitrogen; Glu, blood glucose; TCH, total cholesterol; TG, triglyceride; LDH, lactate dehydrogenase.

*p* value <0.05 was considered statistically significant.

### Variable selection

The Lasso odds ratios plot illustrated linear relationships between features and the target variable, emphasizing association strength (Figure S1A). Shapley additive explanations (SHAP) values from the Extreme Gradient Boosting (XGBoost) model quantified feature impacts, revealing key predictors (Figure S1B). The importance scores derived from both methods were aggregated to generate a comprehensive feature importance ranking on the training set (Figure S1C). Through feature importance ranking and internal validation set area under the curve (AUC) evaluation, we identified the top nine most important features to retain white blood cell (WBC), albumin, blood urea nitrogen (BUN), serum creatinine (Cr), platelet, HCO_3_^−^, blood glucose (Glu), oxygen saturation as measured by pulse oximetry (SpO_2_), and temperature. The multicollinearity analysis of the nine variables revealed that all VIF values were below 5, indicating no significant multicollinearity issues among the variables.

### Model performance comparison

Utilizing these top nine most important features, we developed five different machine learning models, including Decision Tree (DT), LR, Multi-Layer Perceptron (MLP), Support Vector Machine (SVM), and XGBoost. The performance of various classifiers was assessed as depicted in Table 2.

**Table 2:** Predictive performance of the five models for AP associated ARF in internal validation, external validation, and cross-specialty validation cohorts

Cohort	Model	Accuracy	F1 score	AUC (95% CI)	Sensitivity	Specificity	PPV	NPV	Youden _index	Brier _score	Calibration _slope	Calibration_intercept	Calibration _in_the_large
Internal validation set	DT	0.7627	0.7923	0.8437 (0.7325–0.9264)	0.8947	0.7374	0.3953	0.9733	0.6321	0.1383	0.6549	−1.1408	0.6654
	SVM	0.7881	0.8077	0.8203 (0.7177–0.9048)	0.6842	0.8081	0.4062	0.9302	0.4923	0.1547	0.3822	−1.2216	0.6146
	XGBoost	0.8475	0.8593	0.8687 (0.7445–0.9584)	0.7895	0.8586	0.5172	0.9551	0.6481	0.1171	0.6784	−1.2101	0.6723
	LR	0.8220	0.8412	0.9048 (0.8247–0.9674)	0.8947	0.8081	0.4722	0.9756	0.7028	0.1295	1.0406	−1.6774	1.0194
	MLP	0.7966	0.8100	0.8224 (0.6995–0.9184)	0.5789	0.8384	0.4074	0.9121	0.4173	0.1257	0.4864	−1.1657	0.6151
External validation cohort	DT	0.7718	0.7966	0.7586 (0.6963–0.8142)	0.5833	0.8022	0.3231	0.9225	0.3856	0.1417	0.5502	−1.1981	0.6726
	SVM	0.8279	0.8241	0.6928 (0.6065–0.7613)	0.3333	0.9079	0.3692	0.8938	0.2412	0.129	0.3093	−1.0491	0.1060
	XGBoost	0.7911	0.8023	0.7452 (0.6823–0.8056)	0.4028	0.8539	0.3085	0.8983	0.2567	0.1274	0.5305	−1.1121	0.5742
	LR	0.8221	0.8406	0.8717 (0.829–0.9113)	0.7361	0.836	0.4206	0.9514	0.5721	0.0923	0.9590	−0.6615	0.4372
	MLP	0.8375	0.8204	0.702 (0.6254–0.7606)	0.2222	0.9371	0.3636	0.8816	0.1593	0.1312	0.3232	−0.9919	0.0551
Cross-specialty validation cohort	DT	0.5631	0.5825	0.6479 (0.5383–0.7521)	0.5333	0.5753	0.3404	0.7500	0.1087	0.2059	0.5857	−0.718	0.5257
	SVM	0.6214	0.6152	0.5639 (0.4485–0.6785)	0.3000	0.7534	0.3333	0.7237	0.0534	0.2663	0.0833	−0.6884	−0.3173
	XGBoost	0.6602	0.6753	0.8018 (0.6915–0.8881)	0.7000	0.6438	0.4468	0.8393	0.3438	0.1985	0.4654	−0.8092	0.5407
	LR	0.6117	0.6229	0.8557 (0.7758–0.9261)	0.9000	0.4932	0.4219	0.9231	0.3932	0.1801	0.9112	−1.1892	0.7677
	MLP	0.7087	0.7087	0.6968 (0.5812–0.8169)	0.5000	0.7945	0.5000	0.7945	0.2945	0.2465	0.2475	−0.6701	0.3500

Abbreviations: DT, Decision Tree; SVM, Support Vector Machine; XGBoost, extreme gradient boosting; LR, logistic regression; MLP, Multi-Layer Perceptron; AUC, area under the curve; CI, confidence interval; PPV, positive predictive value; NPV, negative predictive value.

**Figure 2:**
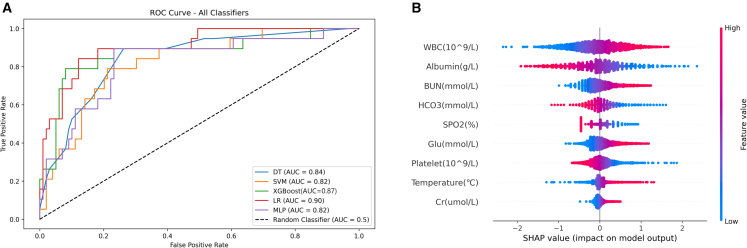
ROC curves of the five models in the internal validation set and important features visualization on the LR model (A) ROC curves of the five models in the internal validation set and (B) the top nine most important features visually presented by the SHAP algorithm based on the LR model. WBC, white blood cell; Cr, serum creatinine; BUN, blood urea nitrogen; Glu, blood glucose.

In the internal validation set, LR excelled with the highest AUC of 0.9048, followed by XGBoost at 0.8687, while DT, MLP, and SVM scored 0.8437, 0.8224, and 0.8203, respectively; LR also exhibited the highest performances with a sensitivity of 0.8947, a negative predictive value (NPV) of 0.9756, and a Youden index of 0.7028 (Table 2), with receiver operating characteristic (ROC) curves confirming its strong discriminative ability (Figure 2A). The LR calibration slope approached 1, indicating optimal calibration; XGBoost and DT exhibited moderate slopes, while SVM and MLP showed lower slopes (Table 2). SHAP analysis was applied to the optimal LR model for ARF in AP, showing feature importance, with red for higher risk and blue for lower. Elevated WBC, BUN, Cr, blood glucose, and temperature, as well as decreased albumin, HCO3^−^, platelet, and SpO_2_ correlated with ARF, with albumin and WBC significantly affecting predictions (Figure 2B).

**Figure 3:**
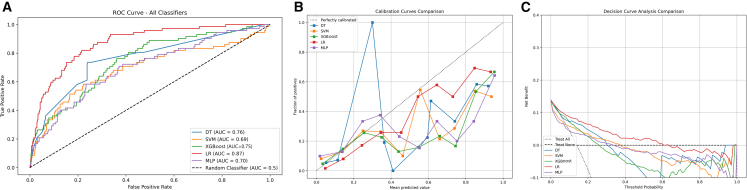
Performance comparison of five models in the external validation cohort (A) ROC curve for each model; (B) calibration plots for each model; (C) DCA curves for each model.

In the external validation cohort, LR achieved the highest AUC of 0.8717, and DT followed with an AUC of 0.7586. XGBoost achieved an AUC of 0.7452, while SVM had the lowest AUC of 0.6928. LR also outperformed the others in sensitivity (0.7361), positive predictive value (PPV) (0.4206), and NPV (0.9514), with the best F1 score (0.8406) and Youden index (0.5721), indicating high stability (Table 2). The ROC curve of LR consistently outperformed the other machine learning algorithms (Figure 3A). The calibration curve demonstrated a satisfactory alignment between the predicted risk of the LR model and the observed risk (Figure 3B). The LR model demonstrated the best overall calibration, with the calibration slope closest to 1, the smallest Brier score, and the calibration intercept closest to zero, supporting its suitability for clinical deployment (Table 2). The decision curve analysis (DCA) depicted in Figure 3C further demonstrated that the LR model had the highest clinical utility. These results suggest that LR remained the most effective classifier based on AUC, while surpassing the other four models across various additional performance parameters.

**Figure 4:**
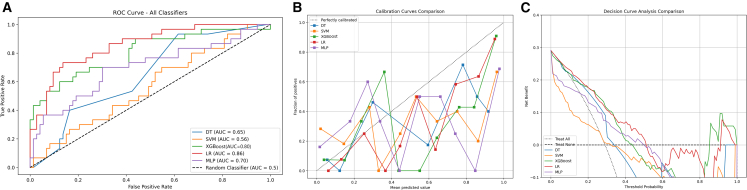
Performance comparison of five models in the cross-specialty validation cohort (A) ROC curve for each model; (B) calibration plots for each model; (C) DCA curves for each model.

In the cross-specialty cohort, the LR model showed the best performance with an AUC of 0.8557, followed by the XGBoost model at 0.8018 and the SVM model at 0.5639 (Table 2). The ROC curve of LR outperformed others in true positive rates (Figure 4A), and DCA indicated LR’s higher net benefit within the 0.1 to 0.5 probability range (Figure 4C). Although the LR model maintained a stable calibration slope close to 1, the calibration curve showed that the LR model overestimated risk (Figure 4B). This deviation may be attributed to the interference of pregnancy-specific physiological changes (e.g., temperature and blood constituents) on prediction stability. Further analysis indicated that seven of the nine key features (WBC, albumin, BUN, Cr, HCO_3_^−^, temperature, and SpO_2_) in the cross-specialty validation cohort exhibited significantly different distributions compared to the derivation cohort, as detailed in Figure S3.

Furthermore, the performance of the LR Model was evaluated within the hypertension and diabetes subgroups of the external validation cohort (Table S1). The LR model demonstrated robust discrimination in both hypertensive and non-hypertensive patients, with AUCs of 0.8302 and 0.8863, respectively. In diabetes, the model had an AUC of 0.7934, while non-diabetes had 0.9086, with a significant difference confirmed (Table S2).

After excluding patients with ARF diagnosed within 2 h of admission, the LR model maintained high and stable AUC values in internal, external, and cross-specialty validation (0.8431, 0.8673, and 0.8397 respectively). The sensitivity analysis yielded similar results to the primary analysis, supporting the validity and robust predictive performance of the models (Table S3).

Therefore, we selected the LR model and developed a web-based prediction model. The 0.30 intervention threshold was selected, and patients with a predicted ARF risk ≥0.30 were classified as high-risk. Clinical practice and patients can access and utilize the application via https://yuwang2009.shinyapps.io/code/. The model testing results were shown in Figure S2.

## Discussion

ARF is the most common type of organ failure in AP and usually the first organ to fail, with both a high incidence and mortality rates.^5^^,^^14^ Therefore, early identification and prediction of the risk of ARF in AP are crucial for improving patient outcomes. To identify high-risk individuals, this study combined multicenter clinical databases with machine learning techniques. By integrating SHAP values and Lasso regression, variable selection was performed from patient gender, age, early admission vital signs and laboratory tests among other variables. This study incorporated temperature, SpO_2_, and seven serum indicators related to ARF into the model features. The LR model based on vital sign and blood biomarkers was developed to enable early identification and monitoring of ARF progression risk in AP. In addition, a cross-specialty validation analysis aimed at examining the performance of this general-population-based model in the APIP group with distinctly different physiological states, thereby exploring a boundary condition of its application.

This study identified the top nine features influencing ARF in AP using SHAP and Lasso regression. Clinical indicators like WBC, HCO_3_^−^, BUN, Cr, blood glucose, and temperature are used in the Acute Physiology and Chronic Health Evaluation II (APACHE II) or Ranson scores to assess acute pancreatitis severity. Hypoalbuminemia was represented as an independent risk factor for persistent organ failure or severity and mortality in AP.^15^^,^^16^ WBC is considered an important biomarker that reflects the degree of inflammation and the body’s response. WBC and albumin have been identified as independent risk factors for respiratory failure in AP.^17^ A previous study reported that both linear and nonlinear imputations of PaO_2_:FiO_2_ from SpO_2_:FiO_2_ demonstrate good performance as long as SpO_2_ < 97%^18^ and SpO_2_:FiO_2_ ratio could be considered as a diagnostic tool of ARDS for early enrollment into clinical trials.^19^ Fei Liu’s study reported BUN could be used to predict the risk of sepsis in AP patients.^20^ And Lin’s study reported higher creatinine levels and lower counts of platelets were associated with an increased risk for ARDS in SAP.^21^ This study indicated that elevated levels of WBC, BUN, Cr, blood glucose, and temperature, along with decreased albumin, HCO_3_^−^, platelet, and SpO_2_, were positively correlated with ARF associated with AP. Clinicians may monitor or intervene to optimize these variables to prevent or limit the development of ARF during the course of AP.

Feature selection is vital for machine learning models with high-dimensional data. This study combined SHAP values and Lasso regression for variable selection, identifying temperature, SpO_2_, and seven serum indicators as input parameters. Previous studies have shown that using SHAP for feature selection can significantly improve the interpretability of models, allowing researchers and clinicians to understand the model’s decision-making process more clearly.^22^ Lasso regression has been demonstrated to automatically choose the features that have the greatest impact on the target variable when dealing with high-dimensional data, thereby improving the predictive power of the model.^23^ The advantage of this combination method lies in the fact that SHAP values can capture the complex nonlinear relationships between features and the target, while Lasso regression can retain important linear relationships and eliminate redundant features, thereby enhancing the interpretability and performance of the model.

Currently, there is a lack of widely applied models for the early prediction of ARF in AP. Although traditional scoring systems such as APACHE II and SOFA scores may assess the prognosis and severity of AP, and LIPS may predict ARDS in patients with AP, incomplete data acquisition and the high complexity of these models limit their practical application for clinicians.^6^^,^^7^^,^^14^^,^^24^ The predictive model used by Zhou’s research employed a single MIMIC database without external validation, which hindered the effective implementation of the predictive model in widely clinical practice.^4^ The prohibitively limited sample size of single-source data and scarcity of positive events rendered stable modeling unfeasible; consequently, this study incorporated databases from the MIMIC database in the United States and a hospital in China. The research maximized the utilization of US data to expand the training set, while concurrently monitoring the model’s generalization capabilities in Chinese populations through a Chinese validation set, providing evidence for parameter tuning. Finally, unbiased evaluations were conducted using an independent external validation set (Chinese data from 2024 to 2025) to better assess the model’s true generalization ability and timeliness. The selected variables were simple, non-invasive, and easily accessible, like demographics, vital signs, SpO_2_, blood counts, and serum markers. The model’s generalizability was validated internally and externally, confirming its applicability across various populations and settings. Sensitivity analysis excluding early-diagnosed ARF further validated the robustness and real predictive ability of LR model. This LR model is recommended for global use in health institutions, including resource-limited areas.

This study applied five machine learning algorithms to medical data for predicting ARF, evaluating the performance of each classifier. The AUC of the LR model achieved the highest values across three validation cohorts, with stability ranging from 0.9048 to 0.8557 (fluctuation <6%).The LR model consistently outperformed others with the highest sensitivities and F1 scores. These results suggested that the LR model has a robust ability to distinguish between positive and negative samples, making it particularly suitable for clinical decision support that requires high stability. Similarly, another study reported the LR algorithm provided the best results and was chosen as the final algorithm to predict macrosomia.^25^

In contrast, a previous study found that single scoring systems demonstrated moderate accuracy in predicting persistent organ failure in patients with AP, with AUC ranges of 0.62–0.84 in the training cohort and 0.57–0.74 in the validation cohort upon admission.^24^ XGBoost has been shown to be effective in predicting ARF and ARDS in studies, particularly when dealing with complex data and in scenarios where feature interactions were significant.^26^^,^^27^ In this study, XGBoost had AUCs of 0.8687, 0.7452, and 0.8018, but a sensitivity drop to 0.4028 in external validation. The LR model outperformed XGBoost in ARF prediction, likely due to its linear feature assumption, making it effective in new data. Thus, we preferred the LR model for its predictive ability and generalization. Future research will further explore and compare the predictive performance of the LIPS score and the LR model for ARF in AP.

To date, the research on the ARF prediction model related to APIP is limited. This study revealed significant physiological differences between the APIP cohort and the general cohort, which explained the systematic overestimation of risk and poor model calibration observed in the APIP cohort. Therefore, the cross-specialty validation showed that the direct application of the model of non-pregnant population to pregnant women could introduce significant prediction bias. Moreover, this study further discussed the robustness of the LR model performance under common complications such as hypertension and diabetes. Subgroup analysis showed that even though blood glucose was included in the LR model, the AUC in diabetic patients was still significantly lower. This indicates that diabetes not only affects the risk of ARF through blood glucose levels but also systematically changes the risk prediction rules through other pathways not fully captured by the model, such as chronic inflammation, endothelial dysfunction, and abnormal immune regulation. Therefore, the performance of the LR model was inherently limited, and it could not be reliably generalized to diabetic populations in its current form. Future research should incorporate tailored recalibration for specific subgroups or develop independent diabetes-specific models. The model showed good discrimination in both hypertensive and non-hypertensive patients, with blood pressure not being a significant predictor, indicating it can be applied robustly across different blood pressure statuses.

Furthermore, the SHAP algorithm and an online computing platform were integrated with the optimal LR classifier for ARF in AP. Clinicians can input nine identified risk factors through a simple form with numeric fields, a process designed to be time-efficient. Upon submission, the tool instantly displays the patient’s individualized probability of developing ARF in AP as a percentage, accompanied by a clear risk stratification (e.g., “low/high risk”). Nine risk factors were described in a hierarchical organization, emphasizing their importance within the model. These visualizations facilitate a deeper understanding of how different features contribute to the model’s decision-making process, offering valuable insights into their relative importance in predicting ARF in AP. Accessible on any internet-connected device, it facilitates point-of-care risk assessment to support clinical decision-making. Based on risk assessment predictions, high-risk patients in the emergency department or routine nursing units could be offered proactive early intervention strategies; these might include enhanced monitoring of key predictive markers, early respiratory support, prompt arterial blood gas analysis, and expedited ICU transfer evaluation—all initiated before clinical deterioration occurs. The effectiveness and interpretability of this method give it broad application potential in future research, especially in complex data environments.

The innovation of this study can be highlighted in three main aspects. First, by integrating the patient’s vital signs with easily obtainable routine laboratory indicators, an interpretable machine learning model was established. This simple and accurate networked predictive model enhanced clinical acceptance with the visualization of feature importance. Second, this study utilized data from two centers in different countries as the derivation and external validation cohorts. The datasets were strictly isolated to eliminate heterogeneity interference between hospitals, with the validation set reflecting the characteristics of the target domain. Model parameters were selected and tuned using historical data from Chinese hospitals to ensure the optimization direction aligned with local clinical practices. Temporal extrapolation validation was employed to assess the model’s generalization capability, supporting its universality and reliability in future clinical environments. Third, the validations of APIP and diabetes primarily revealed boundary conditions of the model. This study provided strong, data-driven evidence for the necessity of developing group-specific models tailored for these unique populations.

### Limitations of the study

However, certain limitations existed in this study. First, although the model validation cohorts provided preliminary insights, they were constrained by sample size and sample proportion imbalance issues—particularly the scarcity of positive events—which might affect the robustness of model performance. Second, this study was retrospective; future prospective studies will include new multicenter patient data as an independent test set to validate the optimized model and continuously monitor feature stability. Additionally, future research could explore the possibility of integrating more data modalities.

## Resource availability

### Lead contact

Requests for further information and resources should be directed to and will be fulfilled by the lead contact, GengYun Sun (2023910208@ahmu.edu.cn).

### Materials availability

This study did not generate new unique reagents.

### Data and code availability

• Data in Medical Information Mart for Intensive Care IV (MIMIC IV) database are available at https://physionet.org/content/mimiciv/2.2/ and https://physionet.org/content/mimic-iv-ed/2.2/.
• Data in the medical record database of First Affiliated Hospital of Anhui Medical University from January 2010 to May 2025 and the Second Affiliated Hospital of Anhui Medical University from January 2016 to December 2023 cannot be made publicly accessible due to hospital regulations. Distributing these data without the necessary consent could potentially breach patient confidentiality and contravene the approval granted by the Ethics Committee for this study.
• Code reported in this paper will be shared on the https://data.mendeley.com/preview/cm4t4225sc?a=ed255215-8358-4da1-933f-ff27f476fabb.
• Any additional information required to reanalyze the data reported in this paper is available from the lead contact upon request.

## Acknowledgments

This study was supported by MIMIC IV, the First Affiliated Hospital of Anhui Medical University, and the second Affiliated Hospital of Anhui Medical University. We appreciate the patients with the acute pancreatitis and their relatives for their willingness to participate and cooperate with the present study.

## Author contributions

Conceptualization, Y.W. and F.L.; data curation, Y.W., F.L., M.T., D.Z., and H.Z.; formal analysis, Y.W., F.L., J.L., and J.X.C.; methodology, Y.W., F.L., J.L., and J.X.C.; investigation, Y.W., F.L., G.Y.S., and X.Y.W.; resources, M.Y.T., D.Z., and H.D.Z.; writing – original draft, Y.W. and F.L.; writing – review and editing, Y.W., F.L., and J.L.; project administration, G.Y.S. and X.Y.W.; supervision, G.Y.S. and X.Y.W. All authors read and approved the final manuscript.

## Declaration of interests

The authors declare that they have no conflict of interest.

## STAR★Methods

### Key resources table

**Table:** 

REAGENT or RESOURCE	SOURCE	IDENTIFIER
**Deposited Data**		

Medical Information Mart for Intensive Care IV (MIMIC IV) database	https://physionet.org/content/mimiciv/2.2/ https://physionet.org/content/mimic-iv-ed/2.2/	Physionet

**Software and algorithms**		

python	https://www.python.org/	Version 3.10.4
scikit-learn	https://scikit-learn.org/	Version 1.6.1
matplotlib	https://matplotlib.org/	Version 3.10.3
xgboost	https://xgboost.readthedocs.io/en/stable/	Version 3.0.2
shap	https://shap.readthedocs.io/en/latest/	Version 0.48.0
R	https://www.r-project.org/	Version 4.5.1
Original code of this study	https://data.mendeley.com/preview/cm4t4225sc?a=ed255215-8358-4da1-933f-ff27f476fabb	N/A

### Experimental model and study participant details

#### Study design and patient selection

This study was a retrospective, multicenter cohort study. Data on acute pancreatitis (AP) were sourced from the MIMIC IV database (https://mimic.mit.edu/docs/iv/) for the years 2008–2019 and from the medical record database of the First Affiliated Hospital of Anhui Medical University in Hefei, China, for the period January 2010 to March 2013, and these data were combined to form the derivation cohort. The external validation cohort comprised AP data from the First Affiliated Hospital of Anhui Medical University, spanning March 2024 to May 2025. Additionally, data on APIP from the First Affiliated Hospital of Anhui Medical University and the Second Affiliated Hospital of Anhui Medical University in Hefei, China, from January 2016 to December 2023 were included for cross-specialty validation.

The inclusion criterion was acute pancreatitis in Adult.

The exclusion criteria were as follows: (1) Patients <18 years old. (2) hospital stay <1 day (3) Repeat ICU admission during an admission.(4)Patients with malignant cancer. (5) Patients diagnosed with chronic respiratory disease. (6) Missing variables of SpO_2_ and WBC.(7) Patients diagnosed with ARF prehospital.

#### Diagnosis and definitions

The diagnosis of AP is according to the 2012 revised Atlanta Criteria for AP, and ARF in AP is defined as a score of 2 or more using the modified Marshall scoring system (arterial oxygen tension divided by fraction of inspired oxygen less than or equal to 300 mmHg).^28^ Or ARF is defined as PaO_2_<60 mmHg on ambient air.^29^

#### Outcomes

The outcome of the study was whether the ARF happened in AP patients within admission to the hospital. Participants who diagnosed with ARF after admission were considered to have a positive outcome, while those who did not were considered to have a negative outcome.

#### Data collection

Data from the MIMIC-IV database were extracted using structured query language within PostgreSQL(version 16, PostgreSQL Global Development Group).The criteria for inclusion in the observation population were that adult patients were confirmed to be diagnosed with AP in ICD-9 or ICD-10.The demographic information, the first vital signs, initial laboratory tests and arterial blood gas analysis, mechanical ventilation, Hospitalization days, ICU admission and in-hospital mortality for each patient were collected from the Emergency Department, hospital or ICU medical record database within 24h before and after hospital admission in MIMIC.

Data of the First Affiliated Hospital of Anhui Medical University and the Second Affiliated Hospital of Anhui Medical University were collected from the Emergency Department, hospital or ICU electronic medical record database. Baseline vital signs, SpO_2_, and laboratory tests were collected at the time of initial diagnosis of AP upon hospital arrival. Initial emergency department laboratory results were prioritized over post-admission in-hospital first tests for primary data analysis, post-admission tests served only for missing data imputation.

#### Ethics approval and consent to participate

The author accessed the MIMIC database after completing the Collaborative Institutional Training Initiative program and was responsible for data extraction and analysis (certification numbers:62927225).

The database was anonymized, eliminating the need for informed consent, which was waived by the Ethics Committees of the First and the second Affiliated Hospital of Anhui Medical University, who approved the study (reference number PJ 2024-07-67,YX2024-181, PJ 2025-02-18). The study followed the STROBE statement guidelines.

As a retrospective study, no animal experiments or clinical trials were performed. Patients’ age, gender, and race information were detailed in Table 1. However, ancestry and ethnicity were not systematically recorded in the source databases, which may influence the conclusions.

The study design complied with the Events Per Variable (EPV) principle.

The total number of enrolled participants, cases of AP patients with ARF, and the final set of predictive variables were presented in the Results section.

### Method details

#### Data preprocessing

The data used in this study underwent comprehensive preprocessing to ensure its quality and suitability for subsequent modeling. Non-numeric entries in the feature matrices were identified and converted into numeric form, with invalid values replaced by missing values (NaN). Logical checks were applied to ensure the plausibility of the data. For instance, age values were restricted to the range of 18–100 years, temperature values to 30°C–45°C, and blood pressure values to 20–300 mmHg. Values outside these logical ranges were treated as missing.

The study’s data underwent thorough preprocessing for quality, excluding features with over 50% missing values.Missing values of continuous variables were filled using the K-Nearest Neighbors (KNN) imputation method, while missing values in categorical variables were imputed with the mode derived from the training set.The Yeo-Johnson transformation was applied to skewed variables, which were then standardized using the *Z* score method. One-hot encoding was utilized for the processing of categorical variables. Synthetic Minority Over-sampling Technique (SMOTE) was applied to the training set to mitigate the negative effects of class imbalance.

#### Variable selection

To assess the importance of features, a combined approach utilizing SHAP values and Lasso regression was adopted within the derivation cohort. SHAP values were computed based on the XGBoost model to quantify the impact of each feature on the model output, while Lasso regression was employed to identify features with the strongest linear relationships to the target variable. The importance scores derived from both methods were combined to generate a comprehensive feature importance ranking. Then, by exhaustive search of the size of the feature subset, the feature subset corresponding to the k value with the highest AUC was finally selected as the optimal feature set. Finally, multicollinearity was addressed by calculating the Variance Inflation Factor (VIF), where variables with a VIF greater than 5 were excluded.

#### Model development and evaluation

The derivation cohort was divided into a training set and an internal validation set. The data of MIMIC-IV and 70% of the Chinese medical records from January 2010 to March 2013 comprised the training set, while the remaining 30%of the Chinese medical records consisted the internal validation set. Patients within the external validation cohort were stratified into four distinct subgroups: hypertension versus non-hypertension and diabetes versus non-diabetes. This work employed five machine learning algorithms for predictive modeling, including LR, SVM, DT, XGBoost, and a customized Artificial Neural Network (ANN) based on MLP. Each classification algorithm underwent hyperparameter tuning through 5-fold cross-validation internally. The LR model utilized the liblinear solver, optimizing the regularization strength C from [0.1, 1] and the penalty type (l1 or l2). The XGB model was trained with a binary classification objective, restricting the maximum tree depth to 3 to prevent overfitting. The SVM employed a Radial Basis Function (RBF) kernel with probability estimates enabled, searching for the optimal regularization parameter C from [1, 10]. For DT, the maximum depth was tuned between values of 3 and 5. Finally, the MLP model was configured with a maximum of 2000 iterations to ensure convergence, optimizing the l2 penalty parameter from [0.001, 0.01] with a fixed initial learning rate of 0.001. The final model for each algorithm was selected based on the highest mean AUC score across validation folds.

The best configurations were retrained on the full training set and evaluated on internal, external and cross-specialty validation cohorts using metrics such as accuracy, F1-score, AUC, sensitivity, specificity, PPV, NPV, and the Youden index. The AUC was used as the primary metric to assess the discriminative ability of each model. The overall ROC curves were plotted for all models, and the model with the highest AUC was selected as the optimal classifier. DCA is a robust method to evaluate the clinical utility of disease diagnostic models. The calibration curve was plotted to assess the consistency between the predicted risk of the models and the observed risk. Combined with all metrics, the predictive performance of each model was analyzed and compared in detail. Bootstrap testing was used to compare AUCs of the optimal model in subgroups. Ultimately, the most optimal model was utilized to develop an online computing platform.

To address the potential bias that predictors might reflect early manifestations of ARF rather than predict it, we performed a sensitivity analysis by excluding patients diagnosed with ARF within 2 h after admission. Model performance was re-evaluated in the internal, external, and cross-specialty validation cohorts.

### Quantification and statistical analysis

Continuous data were tested for normality with the Skewness-Kurtosis test, and summarized as mean (SD) or median (IQR), while categorical variables were reported as frequencies (%). For baseline characteristics analysis, the statistical differences among the three groups were tested with one-way ANOVA or Kruskal-Wallis H for continuous variables and chi-square or Fisher’s exact test for categorical variables, with significance at *p* < 0.05. Analyses were conducted using R (V 4.5.1) and Free Statistics (V 2.2), while machine learning models utilized Sklearn (V1.6.1) and Python (V3.10.4).
