# Supplementary material for: Machine learning model for early prediction of acute respiratory failure in acute pancreatitis: Multicenter validation
Source: iScience. 2026 Jun 18;29(7):116470. doi: 10.1016/j.isci.2026.116470 (PMC13311134; doi:10.1016/j.isci.2026.116470)
Supplement: Document S1. Figures S1–S3 and Tables S1–S3 [file mmc1.pdf]

iScience, Volume *29*

Supplemental information

Machine learning model for early prediction of acute respiratory failure in acute pancreatitis: Multicenter validation

Yu Wang, Feng Lv, MingYang Tao, JiXuan Cui, Dan Zhang, Haodong Zhao, Jun Li, GengYun Sun, and XingYu Wang

Supplemental Figures.

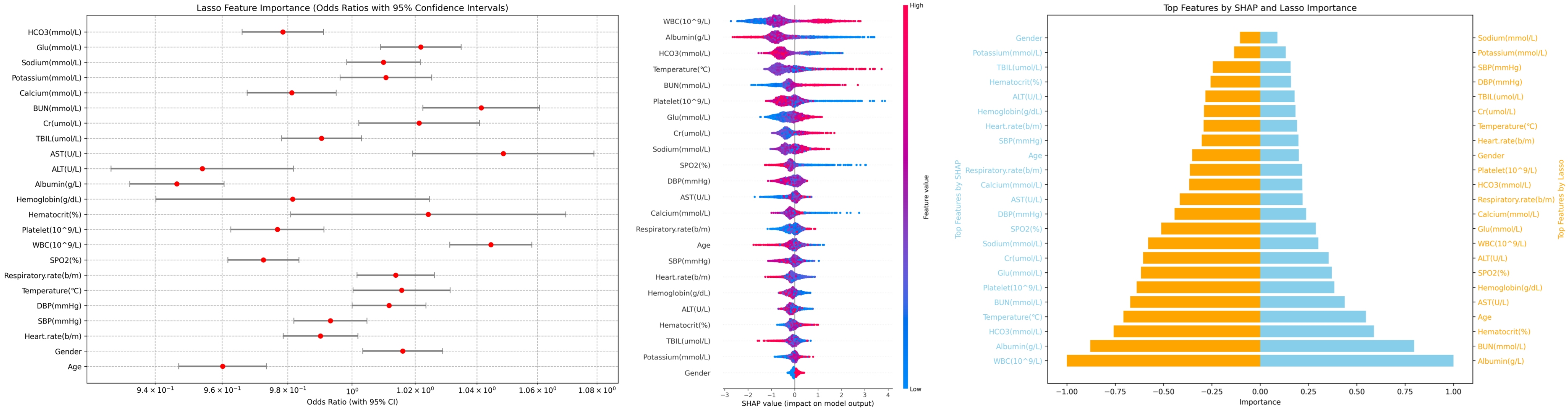

A **B** **C**

Figure S1: Variable selection process of Combined SHAP and Lasso regression.

A)variable selection of Lasso regression.

B)variable selection of SHAP based on the XGBoost model.

C)Combined SHAP and Lasso feature importance plot.

Abbreviations: SBP, Systolic blood pressure; DBP, Diastolic blood pressure; WBC, White blood cell; TBIL,Total Bilirubin; ALT, alanine aminotransferase; Glu,Blood Glucose; AST, aspartate aminotransferase; Cr,Serum creatinine; BUN,Blood Urea Nitrogen.

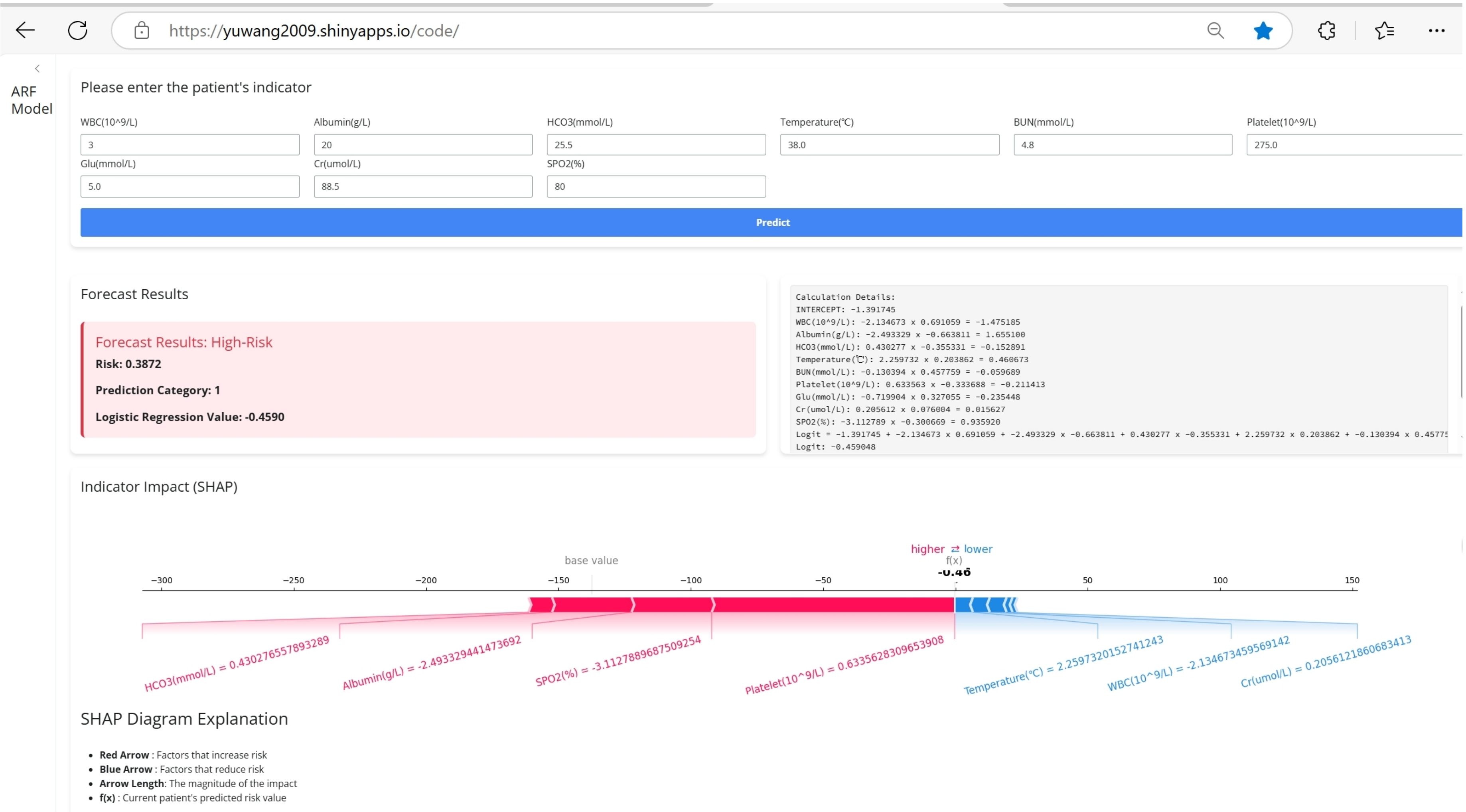

Figure S2: Online computing platform presentation of the optimal LR model.

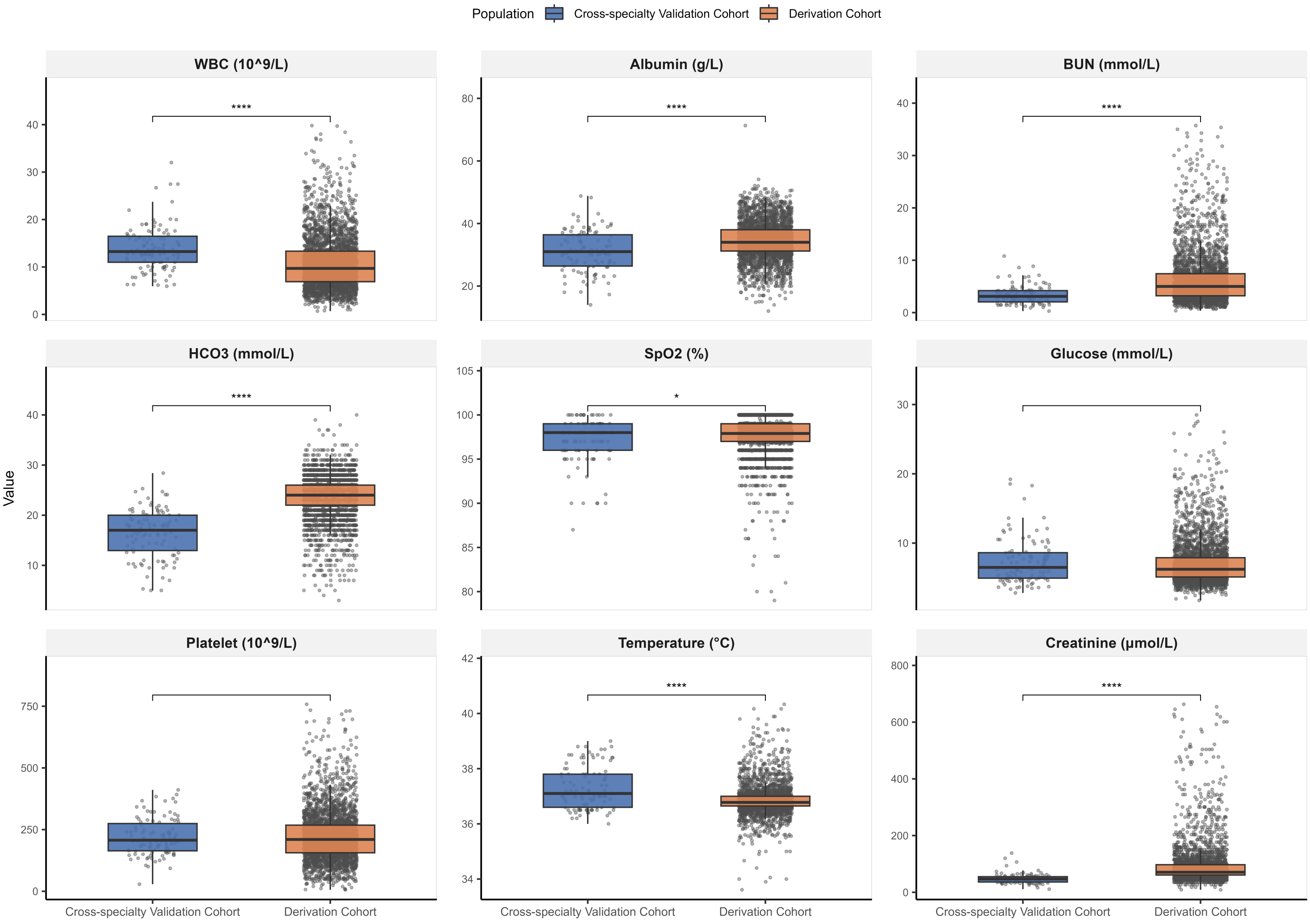

Figure S3: Contrasting distributions of the top 9 most important features between the cross-specialty validation and derivation cohorts.

**** $p < 0.0001$, *** $p < 0.001$, ** $p < 0.01$, * $p < 0.05$

Supplemental Tables

Table S1 Assessing LR model predictive performance across Hypertension and Diabetes subgroups within external validation cohort.

Subgroud	Accuracy	F1 score	AUC(95% CI)	Sensitivity	Specificity	PPV	NPV	Youden_index
Hypertension	0.7353	0.7707	0.8302(0.7395-0.9093)	0.7500	0.7328	0.3261	0.9444	0.4828
Non- Hypertension	0.8530	0.8653	0.8863(0.8346-0.9306)	0.7308	0.8723	0.4750	0.9535	0.6031
Diabetes	0.6783	0.7182	0.7934(0.6799-0.9006)	0.7500	0.6639	0.3103	0.9294	0.4139
Non-Diabetes	0.8797	0.8871	0.9086(0.8631-0.9445)	0.7083	0.9049	0.5231	0.9547	0.6132

Abbreviations: LR, logistic regression;AUC, Area Under the Curve; CI, Confidence Interval; PPV, Positive Predictive Value; NPV, Negative Predictive Value.

Table S2 Comparison of LR Model AUC Across Subgroups with Hypertension and Diabetes in External Validation cohort

Subgroud		AUC	P value
Hypertension	no	0.8863	0.2620
	yes	0.8302	
Diabetes	no	0.9086	0.0420
	yes	0.7934	

AUC, Area Under the Curve;P value<0.05 was considered statistically significant

Table S3. Sensitivity analysis for predictive performance of the five models for AP associated ARF in internal validation , external validation and cross-specialty validation cohorts .

Cohort	Model	Accuracy	F1 score	AUC(95% CI)	Sensitivity	Specificity	PPV	NPV	Youden _index	Brier _score	Calibration _slope	calibration _intercept	Calibration _in_the_large
Internal validation set	DT	0.7895	0.8072	0.7313(0.5944-0.8519)	0.4667	0.8384	0.3043	0.9121	0.3051	0.1760	0.3706	-1.5460	0.9648
	SVM	0.8333	0.8460	0.7562(0.5925-0.8984)	0.6000	0.8687	0.4091	0.9348	0.4687	0.1246	0.3335	-1.2369	0.3313
	XGBoost	0.7719	0.7944	0.7145(0.5397-0.8650)	0.4667	0.8182	0.2800	0.9101	0.2848	0.1531	0.3592	-1.3722	0.7213
	LR	0.8070	0.8321	0.8431(0.6900-0.9630)	0.8000	0.8081	0.3871	0.9639	0.6081	0.1391	0.8516	-1.7246	1.1832
	MLP	0.8070	0.8233	0.7125(0.5407-0.8569)	0.5333	0.8485	0.3478	0.9231	0.3818	0.1758	0.2175	-1.4748	0.5387
External validation cohort	DT	0.8108	0.8276	0.7086(0.6397-0.7757)	0.4386	0.8584	0.2841	0.9227	0.2970	0.1571	0.3560	-1.6013	0.9009
	SVM	0.8765	0.8583	0.7660(0.7028-0.8260)	0.2105	0.9618	0.4138	0.9049	0.1723	0.1026	0.3752	-0.7064	-0.6943
	XGBoost	0.8526	0.8548	0.7862(0.7221-0.8509)	0.3860	0.9124	0.3607	0.9206	0.2983	0.1025	0.5728	-1.069	0.4734
	LR	0.8267	0.8496	0.8673(0.8150-0.9146)	0.7368	0.8382	0.3684	0.9613	0.5750	0.0864	0.9476	-0.9718	0.6657
	MLP	0.8785	0.8631	0.7643(0.7025-0.8267)	0.2456	0.9596	0.4375	0.9085	0.2052	0.1101	0.2961	-1.0232	-0.4505
Cross-specialty validation cohort	DT	0.6214	0.6379	0.6267(0.4960-0.7452)	0.7667	0.5616	0.4182	0.8542	0.3283	0.2793	0.1651	-0.8430	0.7770
	SVM	0.6893	0.6556	0.6279(0.5043-0.7375)	0.2333	0.8767	0.4375	0.7356	0.1100	0.2579	0.1651	-0.3979	-0.9054
	XGBoost	0.7670	0.7670	0.7991(0.6904-0.8910)	0.6000	0.8356	0.6000	0.8356	0.4356	0.1606	0.5588	-0.3671	0.0933
	LR	0.6505	0.6635	0.8397(0.7543-0.9117)	0.9000	0.5479	0.4500	0.9302	0.4479	0.1732	0.8624	-1.0052	0.6586
	MLP	0.7573	0.7150	0.7374(0.6223-0.8375)	0.2667	0.9589	0.7273	0.7609	0.2256	0.2327	0.2452	-0.1201	-0.9906

Abbreviations: DT, Decision Tree; SVM, Support Vector Machine; XGBoost, extreme gradient boosting; LR, logistic regression; MLP, Multi-Layer Perceptron; AUC, Area Under the Curve; CI, Confidence Interval; PPV, Positive Predictive Value; NPV, Negative Predictive Value.
